# Effects of Tramadol on Substantia Gelatinosa Neurons in the Rat Spinal Cord: An *In Vivo* Patch-Clamp Analysis

**DOI:** 10.1371/journal.pone.0125147

**Published:** 2015-05-01

**Authors:** Hiroyuki Yamasaki, Yusuke Funai, Tomoharu Funao, Takashi Mori, Kiyonobu Nishikawa

**Affiliations:** Department of Anesthesiology, Osaka City University Graduate School of Medicine, Osaka, Japan; University of Texas at Dallas, UNITED STATES

## Abstract

Tramadol is thought to modulate synaptic transmissions in the spinal dorsal horn mainly by activating µ-opioid receptors and by inhibiting the reuptake of monoamines in the CNS. However, the precise mode of modulation remains unclear. We used an *in vivo* patch clamp technique in urethane-anesthetized rats to determine the antinociceptive mechanism of tramadol. *In vivo* whole-cell recordings of spontaneous inhibitory postsynaptic currents (sIPSCs) and spontaneous excitatory postsynaptic currents (sEPSCs) were made from substantia gelatinosa (SG) neurons (lamina II) at holding potentials of 0 mV and -70 mV, respectively. The effects of intravenous administration (0.5, 5, 15 mg/kg) of tramadol were evaluated. The effects of superfusion of tramadol on the surface of the spinal cord and of a tramadol metabolite (M1) were further analyzed. Intravenous administration of tramadol at doses >5 mg/kg decreased the sEPSCs and increased the sIPSCs in SG neurons. These effects were not observed following naloxone pretreatment. Tramadol superfusion at a clinically relevant concentration (10 µM) had no effect, but when administered at a very high concentration (100 µM), tramadol decreased sEPSCs, produced outward currents, and enhanced sIPSCs. The effects of M1 (1, 5 mg/kg intravenously) on sEPSCs and sIPSCs were similar to those of tramadol at a corresponding dose (5, 15 mg/kg). The present study demonstrated that systemically administered tramadol indirectly inhibited glutamatergic transmission, and enhanced GABAergic and glycinergic transmissions in SG neurons. These effects were mediated primarily by the activation of μ-opioid receptors. M1 may play a key role in the antinociceptive mechanisms of tramadol.

## Introduction

Tramadol is widely used as an analgesic for the treatment of postoperative, cancer, or chronic neuropathic pain [1, 2]. Its analgesic effects have been reported following its systemic administration in rat acute and chronic pain models [3, 4]. Two main mechanisms are thought to contribute to the antinociceptive action of tramadol in the central nervous system and spinal cord: the activation of μ opioid receptors [5, 6] and the inhibition of the neuronal uptake of noradrenaline and serotonin (5-HT) [7]. Tramadol itself acts on several ion channels and receptors, including sodium channels, GABA_A_ receptors, NMDA receptors, and nicotinic acetylcholine receptors [8–10]. These findings suggest that sensory nociceptive transmission in the spinal cord may be modulated in several different ways.

The superficial dorsal horn, specifically the substantia gelatinosa (SG) lamina II of the spinal cord, is involved in transmission of peripheral pain signals to the central nociceptive field [11, 12]. SG neurons receive noxious information by glutamatergic synaptic inputs from peripheral Aδ and C-afferent fibers [13]. They also receive abundant inhibitory synaptic inputs from GABAergic and glycinergic interneurons [14], thus, they are modulated by the descending inhibitory system. A recently developed “*in vivo* patch-clamp” technique for the spinal dorsal horn has enabled the assessment of the actions of systemically administered drugs on synaptic activity in SG neurons.

Opioid receptors are located abundantly in pain pathways and in the descending inhibitory system. Tramadol and its metabolite O-desmethyl tramadol (M1) have the opioid analgesic properties. M1 has higher affinity and efficacy for opioid μ receptors than tramadol [15]. A previous electrophysiological study using rat spinal cord slices demonstrated that M1 induced outward currents by activating μ-receptors in SG neurons [16], which suggested that the marked hyperpolarization of SG neurons may inhibit nociception. A previous study using microdialysis reported systemic tramadol-induced increases in noradrenaline and 5-HT in the spinal dorsal horn, indicating the involvement of descending inhibitory pathways in its antinociceptive mechanisms [3]. These findings demonstrated the importance of understanding how systemic tramadol modulates synaptic transmission at SG neurons in the spinal cord *in vivo*.

In the present study, we used the *in vivo* patch-clamp technique to record spontaneous excitatory post synaptic currents (sEPSCs), spontaneous inhibitory post synaptic currents (sIPSCs), and slow membrane currents from SG neurons in the rat spinal dorsal horn.

## Materials and Methods

All experimental procedures were approved by the Ethics Committee on Animal Experiments at Osaka City University (approval number: 13044) and performed according to the Guiding Principles for the Care and Use of Animals recommended by the Physiological Society of Japan. All efforts were made to minimize the number of animals used in the experiments.

### Behavioral tests

Behavioral testing was conducted in a silent room away from the colony room, in daylight at a standard temperature (24 ± 1°C). Six male rats, aged 6 weeks, were included in this protocol. The rats were allowed to acclimate to the test location for 1 hour before the experiment. Rats were placed onto a perforated metal mesh platform, and mechanical stimuli were delivered to the hindpaw using the Dynamic Plantar Aesthesiometer (37450, Ugo Basile, Comerio, Italy). This instrument, located under the platform, raised a straight metal filament, 0.5 mm in diameter, until it contacted with the plantar surface of the hindpaw and exerted a gradually increasing upward force (from 1 to 50 g over 20 seconds) until the paw was withdrawn. The mechanical withdrawal threshold was quantified for the right paw from an average of 5 Aesthesiometer trials. Control measurements were obtained using normal saline and then 0.5 mg/kg of tramadol, 5 mg/kg of tramadol, and 4 μg/kg of naloxone were administered intraperitoneally and sequentially to the same rat. Pain thresholds were evaluated twenty minutes after the administration of tramadol or normal saline, and ten minutes after the administration of naloxone.

### *In vivo* patch clamp recordings

The experimental method was previously described at length [17, 18]. Briefly, 96 male Sprague-Dawley rats (6–9 weeks of age, 210–380 g) were anesthetized with urethane (1.2–1.5 g/kg intraperitoneally), and the left femoral vein was cannulated for drug administration. The lumbar spinal cord was exposed by thoracolumbar laminectomy at the level from Th13 to L2, and the rat was then placed in a stereotaxic apparatus (Model ST-7; Narishige, Tokyo, Japan). Spinalized rats underwent a laminectomy at the cervical level to allow right-sided cord hemisection to disturb descending inhibitory pathways ipsilateral to the recording side. The dura was opened and then the dorsal root that enters the spinal cord above the level of the recording sites was lifted so that a recording electrode could be inserted into the SG from the surface of the spinal cord. The surface of the spinal cord was irrigated with 95% O_2_-5% CO_2_-equilibrated Krebs solution (15 ml/min; NaCl, 117; KCl, 3.6; CaCl2, 2.5; MgCl2, 1.2; NaH2PO4, 1.2; glucose, 11; NaHCO3, 25 mM) through glass pipettes at 36.5°C. The pia-arachnoid membrane was cut to allow the patch electrode to enter the spinal cord.

Whole-cell voltage-clamp recordings were conducted from SG neurons with a patch electrode with a tip resistance of 8–12 MΩ and filled with a pipette solution containing the following (in mM): 110 Cs_2_SO_4_, 5 TEA-Cl, 0.5 CaCl_2_, 2 MgCl_2_, 5 EGTA, 5 HEPES, and 5 Mg-ATP, in the 0mV voltage-clamp mode to observe GABAergic and/or glycinergic IPSCs, or 136 K-gluconate, 5 KCl, 0.5 CaCl_2_, 2 MgCl_2_, 5 EGTA, 5 HEPES, and 5 Mg-ATP, mainly in the -70 mV voltage clamp mode to observe EPSCs and slow membrane currents.

SG neurons were recorded at a depth of 50–150 μm from the dorsal surface of the spinal cord at the L2-L4 level [17]. Signals were collected with a patch clamp amplifier (Axopatch 200B; Molecular Devices, Union City, CA). Data were digitized using an analog-to-digital converter (Digidata1321A, Molecular Devices, Union City, CA), stored on a personal computer using a data acquisition program (Clampex version 8.0; Molecular Devices, Union City, CA), and analyzed using a special software package (Clampfit version 4.1; Molecular Devices, Union City, CA). The frequencies, amplitudes, and synaptic charge transfers of spontaneous IPSCs (sIPSCs) and EPSCs (sEPSCs) were measured automatically using MiniAnalysis software (Synaptosoft, Decatur, GA). The amplitude of each postsynaptic current was defined as the amount from the initial inflection point (not from the baseline) to the peak. The validity of this method was confirmed by a visual analysis of all traces on a fast-time scale before they were accepted for further investigations.

Drugs for intravenous administration were injected via the left femoral vein. The amounts of systemically-administered tramadol were 0.5, 5, and 15 mg/kg. The dosage of intravenous tramadol applied under *in vivo* conditions was determined based on clinically relevant doses. Drugs for spinal applications were diluted in Krebs solution and superfused onto the surface through inlet and outlet glass pipettes by exchanging solutions at a constant perfusion rate and temperature. The interval necessary for the test drug to reach the surface of the spinal cord was 5 seconds. The amounts of tramadol dissolved in Krebs solution were 10 and 100 μM. At the end of the experiments, the rats were euthanized by the administration of an overdose of pentobarbital.

The following drugs were purchased from Sigma Aldrich Japan (Tokyo, Japan): tramadol hydrochloride, M1, naloxone hydrochloride dihydrate, bicuculline, strychnine, and CNQX.

### Statistical analysis

The total area of IPSCs and EPSCs over a 10 second period was measured to reflect the ongoing synaptic charge transfer, which indicated the intensity of each synaptic current. Modification of areas of sEPSCs and sIPSCs were assessed as the percentage of control. We defined neurons as being sensitive to a particular drug when the synaptic charge transfer was altered by more than ± 15% from the control. All numerical data were expressed as the mean ± SEM. Significance was defined as *p* < 0.05 by a one-way analysis of variance (ANOVA) with Dunnett’s post hoc test for withdrawal threshold data and the paired *t*-test for the absolute values of the amplitude, frequency, or synaptic charge transfer of IPSCs and EPSCs. Significantly different values were indicated by asterisks in the figures or by the Kolmogorov–Smirnov test for their probabilities. In all cases, n referred to the number of neurons tested. Sample size calculation was based on the values of synaptic charge transfer in our previous study [19]. Five cells were required for each experiment (α = 0.05 and β = 0.2).

## Results

### Analgesic effects of systemic tramadol on mechanical nociception

**Fig 1:**
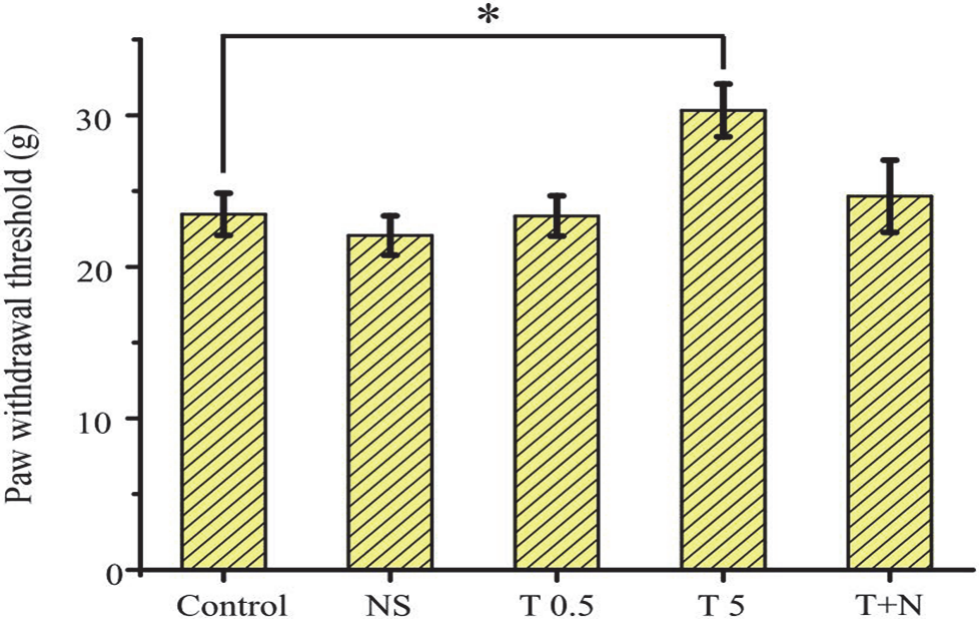
Anti-nociceptive effects of systemic tramadol. Paw withdrawal thresholds (PWT) were sequentially evaluated before (control) and after the intraperitoneal administration of normal saline (NS), tramadol 0.5 mg/kg (T 0.5), 5 mg/kg (T 5), and naloxone 4 μg/kg (T+N) in this sequential order to the same rats. The PWT was elevated with tramadol 5 mg/kg (*n* = 6) and was returned to the control level by naloxone. Data are the mean ± SEM. Asterisks indicate a significant difference from the control (*p < 0.05) (one-way analysis of variance and Dunnett’s post hoc test).

We evaluated the paw withdrawal response to noxious mechanical stimuli for behavioral analysis in conscious rats to determine the analgesic effects of systemic tramadol (Fig 1). The mechanical withdrawal threshold of the control was 23.5 ± 1.4 g. An intraperitoneal injection of normal saline (NS) and a small dose of tramadol (T0.5; 0.5 mg/kg) had no effect on this threshold (22.1 ± 1.3 g; *p* > 0.05, 23.4 ± 1.3 g; *p* > 0.05, respectively), whereas tramadol at a dose of 5 mg/kg (T5) significantly increased the mechanical withdrawal threshold (30.3 ± 1.7 g; *p* < 0.05). This effect was almost reversed by an intraperitoneal injection of 4 μg naloxone (T+N) (24.7 ± 2.4 g; *p* > 0.05). All the animals awoke and showed activity with no obvious sedation during the experimental period, even after the administration of 5 mg/kg tramadol.

### Recording synaptic currents from SG neurons

**Fig 2:**
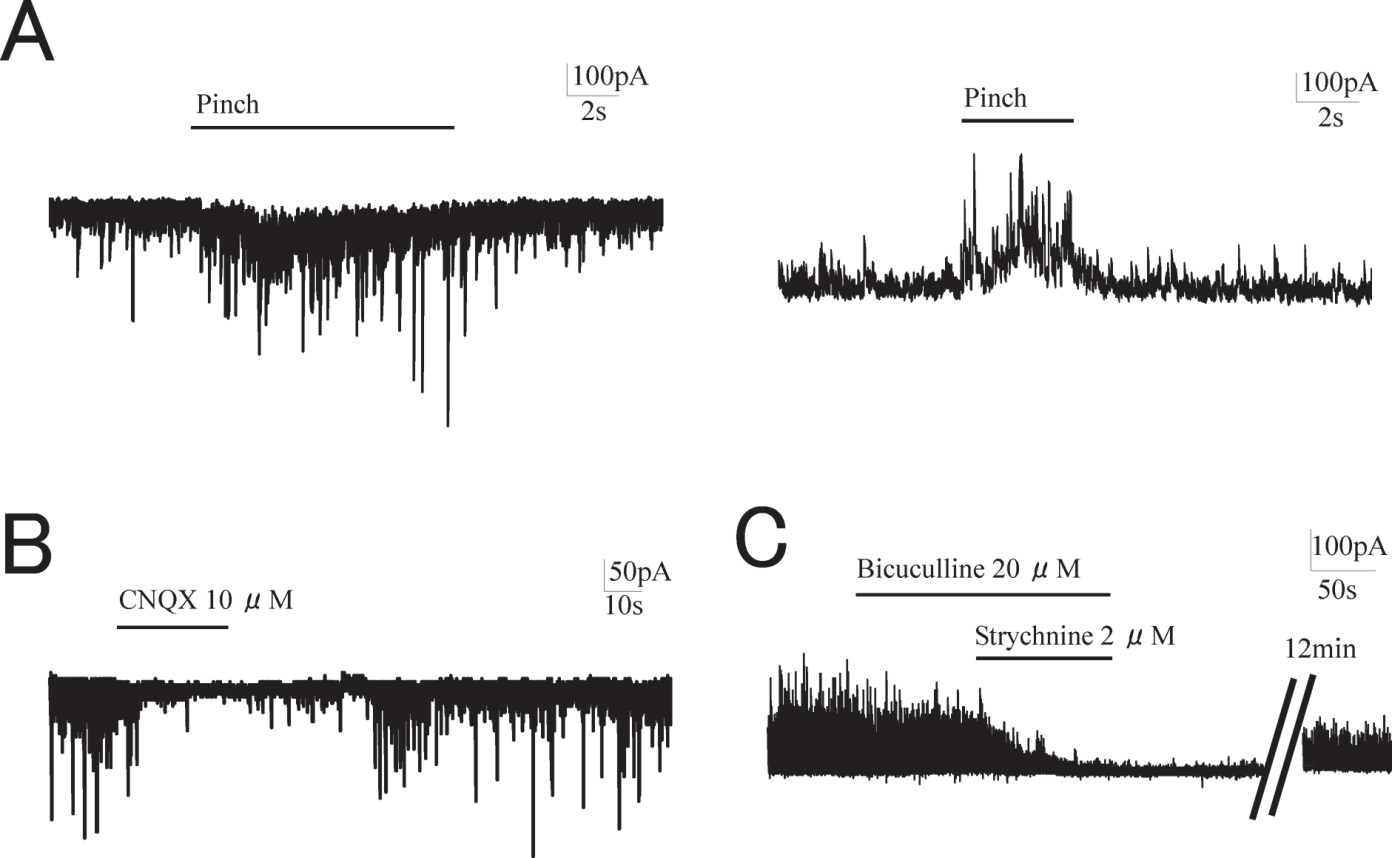
The characteristics of synaptic currents in substantia gelatinosa neurons. (A) The identification of SG neurons was made by the response of the EPSCs and IPSCs to a pinch stimuli applied to rat hindpaw. (B) The perfusion of CNQX (10 μM) on the spinal cord immediately abolished spontaneous excitatory postsynaptic currents (sEPSCs). (C) Coadministration of bicuculline (20 μM) and strychnine (2 μM) in the perfusion solution diminished spontaneous inhibitory postsynaptic currents (sIPSCs).

We used a previously described *in vivo* patch-clamp technique [19] for successful recording of EPSCs and IPSCs from spinal dorsal horn neurons. As this technique is blind patch-clamp recording, the sensory SG neurons were identified by the response of the EPSCs and IPSCs to a touch and pinch stimuli applied to rat hindpaw [17, 20] (Fig 2A). In SG neurons, the EPSCs and the IPSCs exhibited stable activity without noxious stimuli. These were so-called spontaneous currents. The effects of tramadol were determined on spontaneous EPSCs (sEPSCs) and spontaneous IPSCs (sIPSCs) since the spontaneous currents were considered to represent synaptic activity of SG neurons. The sEPSCs and sIPSCs exhibited sufficient amplitudes for evaluations at holding potentials of -70 mV and 0 mV, respectively, using each specific pipette solution. The outward currents were analyzed under the same conditions as those used for the EPSCs [16].

*In vivo* patch-clamp recordings were performed in rats aged 6–9 weeks. Our preliminary experiments that compared spontaneous IPSCs between cells obtained from 5–6 week and 7–9 week-aged rats revealed no significant difference in the frequency (5–6 week rats, 46.9 ± 5.4 Hz, *n* = 30; 7–9 week rats, 49.8 ± 4.9 Hz, *n* = 30; *p* > 0.05) or amplitude (5–6 week rats, 28.7 ± 2.6 pA, *n* = 30; 7–9 week rats, 32.7 ± 2.9 pA, *n* = 30; *p* > 0.05). These observations suggested that the difference in a few weeks of age would not largely affect the results of our experiments.

We confirmed the characteristics of sEPSCs and sIPSCs by superfusion of CNQX (an AMPA/Kainate antagonist), bicuculline (a GABA_A_ receptor antagonist), and strychnine (a glycine receptor antagonist) onto the spinal cord. The sEPSCs rapidly disappeared almost completely after 10 μM CNQX superfusion and then recovered following washout of the solution (Fig 2B). The synaptic charge transfer of sEPSCs was 4 ± 2% of the control in the presence of 10 μM CNQX (*n* = 5, *p* < 0.05). Superfusion of 20 μM bicuculline decreased the sIPSCs and coadministration of 2 μM strychnine with bicuculline almost completely eliminated them (Fig 2C). The synaptic charge transfer of sIPSCs was 52 ± 11% of the control in the presence of strychnine and was further reduced to 5 ± 3% in the presence of bicuculline and strychnine (n = 5, *p* < 0.05). These observations indicated that the sEPSCs were mostly mediated via AMPA/Kainate receptors, while the sIPSCs were mediated by both GABA_A_ and glycine receptors.

### Systemic tramadol inhibited EPSCs

**Fig 3:**
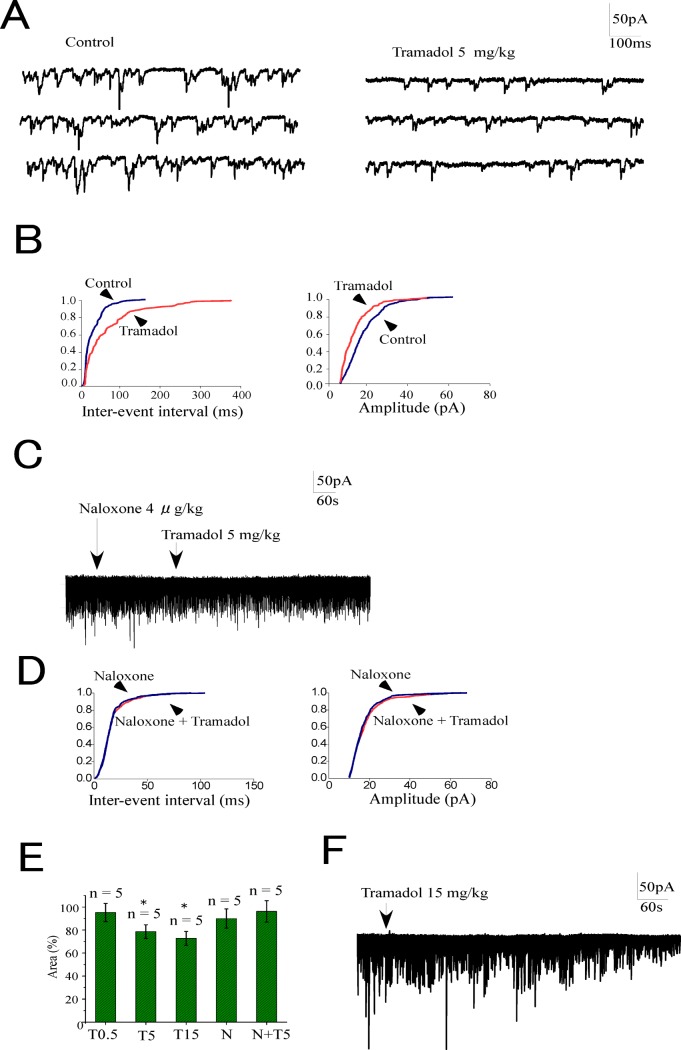
Effects of systemic tramadol on spontaneous excitatory currents (sEPSCs) in substantia gelatinosa neurons. (A) Systemic tramadol (5 mg/kg) inhibited sEPSCs. (B) Cumulative probability plots obtained from the trace in panel A showed a prolongation of inter-event intervals and a decrease in amplitude induced by tramadol. (C) Systemic tramadol (5 mg/kg) had no effect on sEPSCs 5 minutes after the administration of naloxone (4 μg/kg). (D) Cumulative probability plots obtained from the trace in panel C showed that systemic tramadol did not change inter-event intervals and amplitude when naloxone was used as a pretreatment. (E) Summary of the effects of tramadol on sEPSCs. The ordinate represents synaptic charge transfers of sEPSCs as percentages of the control (before the administration of drugs). Tramadol inhibited sEPSCs at doses higher than 5 mg/kg (*p < 0.05). Neither naloxone (4 μg/kg) itself nor tramadol (5 mg/kg) after naloxone had any effect on sEPSCs. Data are the mean ± SEM. Asterisks indicate a significant difference from the control (*p < 0.05) (paired *t*-test). (F) Tramadol did not induce outward currents even at a high dosage (15 mg/kg). Abbreviations: T0.5, tramadol 0.5 mg/kg; T5, 5 mg/kg; T15, 15 mg/kg; N, naloxone

We examined the effects of systemically administered tramadol on spontaneous EPSCs (sEPSCs). Intravenous tramadol at doses of 0.5, 5, and 15 mg/kg inhibited sEPSCs in 2 out of 5, 3 out of 5, and 4 out of 5 neurons, respectively. The trace in Fig 3A shows that sEPSCs decreased following the intravenous injection of 5 mg/kg tramadol. These inhibitory effects reached a maximum 5–15 min after the injections at all doses tested. The cumulative event distribution curves obtained from the recordings in Fig 3A showed that systemic tramadol (5 mg/kg) shifted the curve toward a lengthening of the inter-event interval and a decreasing amplitude of the EPSCs (Fig 3B). Tramadol at a dose of 5 mg/kg had no effect on sEPSCs when naloxone 4 μg/kg was administered intravenously 5 min before tramadol (Fig 3C and 3D). Naloxone alone had no effect on EPSCs.

We quantified the effects of tramadol on EPSCs by obtaining the synaptic charge transfers of EPSCs by calculating the area under the EPSCs for a 10-sec period and normalizing to the level before drug administrations (control). A small dose of intravenous tramadol (0.5 mg/kg) did not significantly decrease sEPSCs (T0.5) (95 ± 8%, *n* = 5). Tramadol at doses over 5 mg/kg decreased the synaptic charge transfer of sEPSCs to 79 ± 6% of the control (n = 5) at 5 mg/kg (T5) and 73 ± 6% (*n* = 5) at 15 mg/kg (T15). The pretreatment of systemic naloxone abolished tramadol-induced decreases in EPSCs (N+T5) (96 ± 9%, *n* = 5) (Fig 3E). Obvious outward currents were not observed after intravenous tramadol even at a high dose (Fig 3F).

### Systemic tramadol enhanced IPSCs

**Fig 4:**
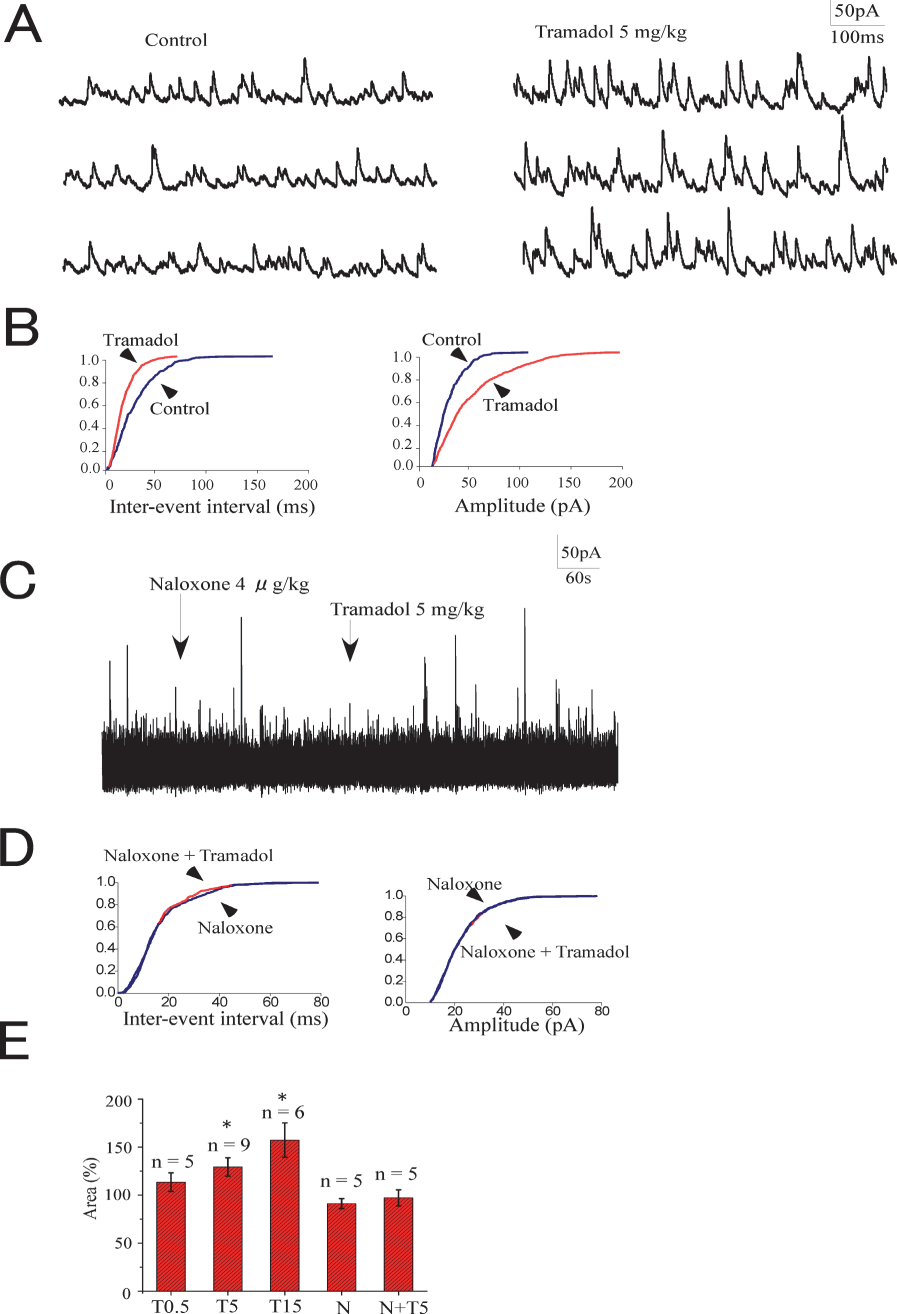
Effects of systemic tramadol on sIPSCs in substantia gelatinosa neurons. (A) Systemic tramadol (5 mg/kg) increased sIPSCs. (B) Cumulative probability plots obtained from the trace in panel A showed the shortening of inter-event intervals and increase in amplitude induced by tramadol. (C) Systemic tramadol (5 mg/kg) had no effect on sIPSCs 5 minutes after the administration of naloxone (4 μg/kg). (D) Cumulative probability plots obtained from the trace in panel C showed that systemic tramadol did not change inter-event intervals and amplitude when naloxone was used as a pretreatment. (E) Summary of tramadol effects on sIPSCs. The ordinate represents synaptic charge transfers of sIPSCs as percentages of the control (before the administration of drugs). Tramadol increased sIPSCs at doses higher than 5 mg/kg (*p < 0.05). Neither naloxone (4 μg/kg) itself nor tramadol 5 mg/kg after naloxone had any effect on sIPSCs. Data are the mean ± SEM. Asterisks indicate a significant difference from the control (*p < 0.05) (paired *t*-test). Abbreviations: T0.5, tramadol 0.5 mg/kg; T5, 5 mg/kg; T15, 15 mg/kg; N, naloxone

We determined the effects of systemically administered tramadol on spontaneous IPSCs (sIPSCs) in SG neurons. An enhancement of sIPSCs by intravenously administered tramadol was detected in 2 out of 5, 6 out of 9, and 5 out of 6 neurons at doses of 0.5 (T0.5), 5 (T5), and 15 (T15), respectively. The trace in Fig 4A shows an increase in sIPSCs after the administration of tramadol (5 mg/kg).

The enhancement observed in sIPSCs following systemic tramadol administration reached a maximum approximately 5–10 min after the injection. Tramadol shifted the cumulative event distributions of sIPSCs to shorten the inter-event interval and increase their amplitude (Fig 4B). Fig 4C and 4D show that 5 mg/kg tramadol had no effect on spontaneous IPSCs when naloxone 4 μg/kg was administered intravenously 5 min before tramadol (N+T5). Naloxone alone had no effect on sIPSCs. The inhibitory synaptic charge transfers of sIPSCs were significantly enhanced by systemic tramadol at doses of 5 and 15 mg/kg. These enhancements were 114 ± 9% (*n* = 5), 129 ± 10% (*n* = 9), and 157 ± 18% (*n* = 6) of the control at doses of 0.5, 5, and 15 mg/kg, respectively (Fig 4E). The pretreatment with systemic naloxone abolished tramadol-induced increases in sIPSCs (97 ± 8%, *n* = 5) (Fig 4E).

### The effects of tramadol superfusion on synaptic transmission in SG neurons

**Fig 5:**
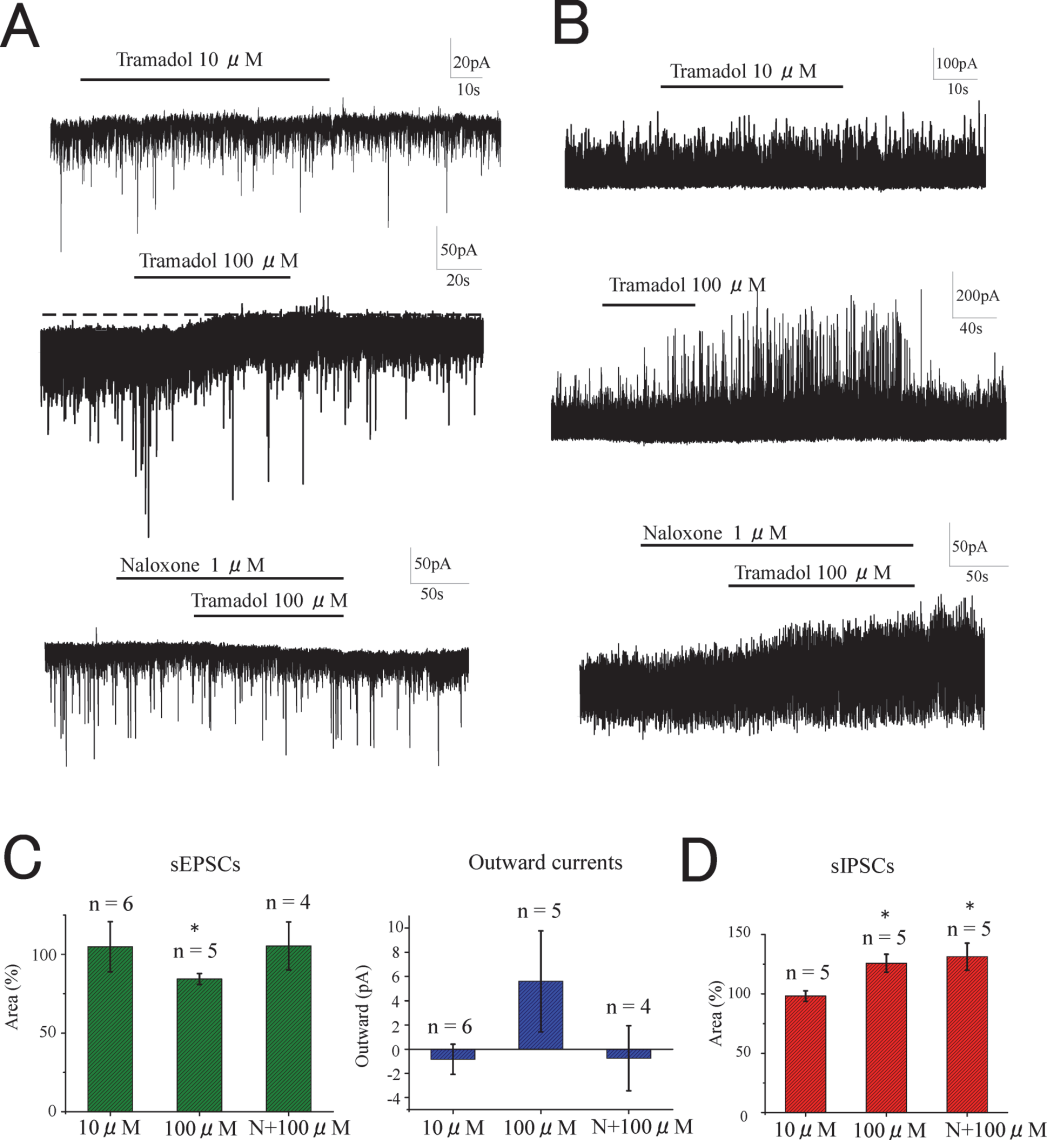
Effects of tramadol superfusion on sEPSCs and sIPSCs in substantia gelatinosa neurons. Superfusion of tramadol at clinically relevant concentration (10 μM) onto the surface of the spinal cord had no effect on sEPSCs (A upper trace). At high concentration (100 μM) superfusion of tramadol inhibited sEPSCs and induced outward currents (A middle trace). In the presence of naloxone 1 μM, superperfusion of tramadol 100 μM had no effect on sEPSCs (A lower trace). Superfusion of tramadol had no effect on sIPSCs at clinically relevant concentration (10 μM) (B upper trace), but enhanced sIPSCs at high concentration (100 μM) (B middle trace). The enhancement at high concentration was observed even in the presence of naloxone (B lower trace). These effects were summarized in (C). Data are the mean ± SEM. Asterisks indicate a significant difference from the control (*p < 0.05) (paired *t*-test).

We observed the direct actions of tramadol on the spinal cord by applying concentration-adjusted tramadol-containing Krebs solutions by superfusion on the surface of the spinal cord for 3 min during the recordings of sEPSCs and sIPSCs. The superfusion of 10 μM tramadol did not change the activity of sEPSCs (synaptic charge, 105 ± 16% of the control, *n* = 5, *p* > 0.05) (Fig 5A upper trace,Fig 5C). In contrast, higher concentrations (100 μM) of tramadol produced outward currents (5.6 ± 4.2 pA) and decreased the activity of sEPSCs (synaptic charge, 84 ± 3% of control, *n* = 5, *p* < 0.05) (Fig 5A middle trace,5C). The decreases in sEPSCs and the outward currents induced by a high concentration of tramadol (100 μM) were not observed when 1 μM naloxone was pre-perfused (synaptic charge, 105 ± 15% of the control, *n* = 5) (Fig 5A lower trace and 5C). Tramadol (10 μM) had no effect on spontaneous IPSCs (synaptic charge, 98 ± 4% of the control, *n* = 5, *p* > 0.05) (Fig 5B upper trace,5D) while a higher concentration of tramadol (100 μM) enhanced sIPSCs (synaptic charge, 126 ± 8% of the control, *n* = 5, *p* < 0.05) (Fig 5B middle trace,5D). However, tramadol (100 μM) enhanced sIPSCs even when naloxone was preperfused (synaptic charge, 131 ± 11% of the control, *n* = 5, *p* < 0.05) (Fig 5B lower trace,5D).

### The effects of O-desmethyltramadol (M1) on synaptic transmission in SG neurons

The results presented thus far for the present study indicate the existence of a discrepancy in the modulations of tramadol on synaptic transmissions between intravenous tramadol and superfusion. Intravenous tramadol modulated synaptic activities in SG neurons, while tramadol superfusion at a clinically relevant concentration (10 μM) had no effect.

**Fig 6:**
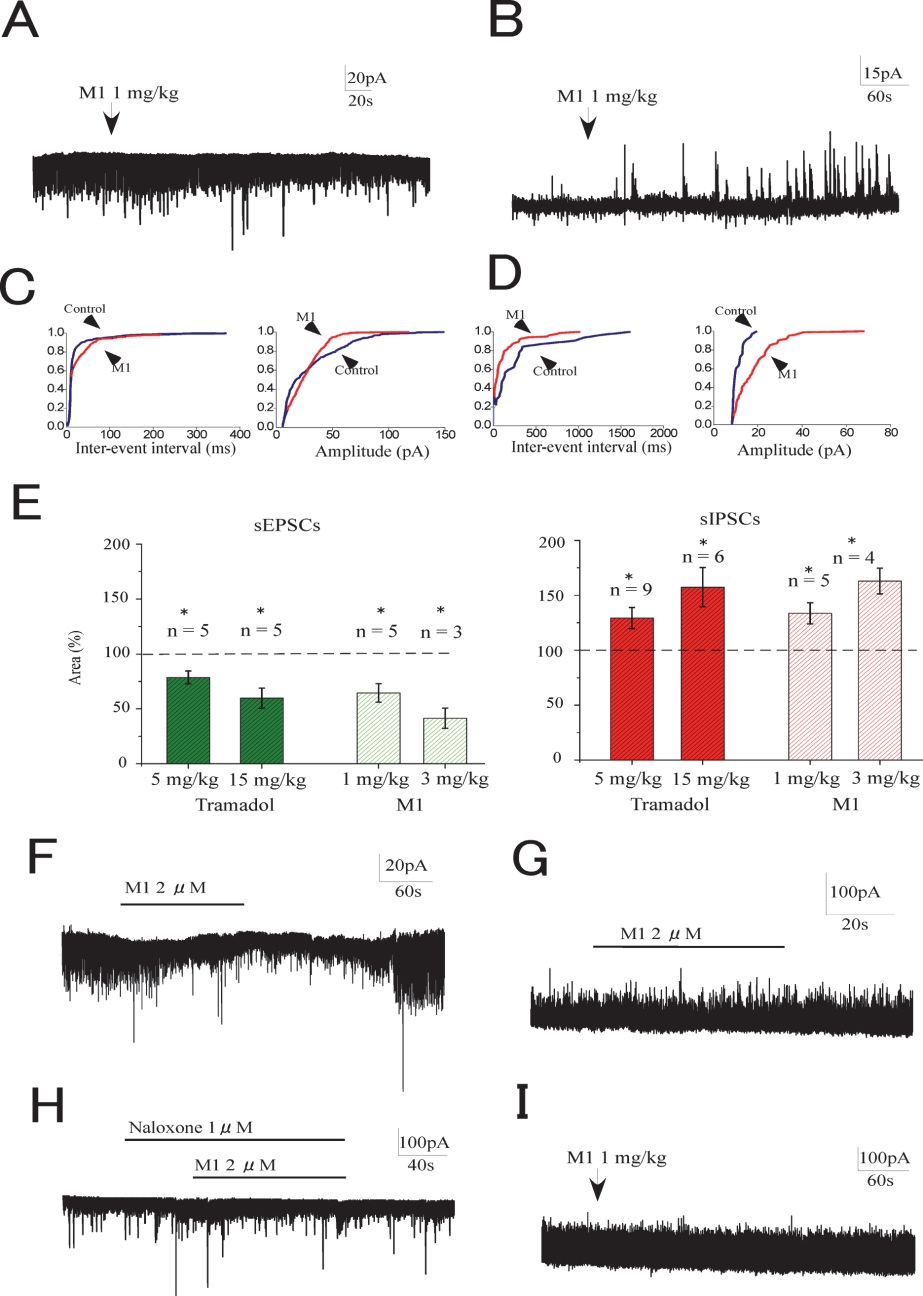
Effects of O-desmethyltramadol (M1) on sEPSCs and sIPSCs in substantia gelatinosa neurons. Systemically administered M1 (1 mg/kg) inhibited sEPSCs (A) and enhanced sIPSCs (B). (C) shows cumulative probability plots obtained from the trace in panel A. (D) shows cumulative probability plots obtained from the trace in panel B. The inhibition of sEPSCs (E left) and the enhancement of sIPSCs (E right) by M1 (1. 3 mg/kg) were comparable with those of tramadol (5, 15 mg/kg). The superfusion of M1 (2 μM) decreased sEPSCs (F) but no effect on sIPSCs (G). The superfusion of M1 had no effect on sEPSCs when naloxone was preperfused (1 μM) (H). In spinalized rat, systemic M1 (1 mg/kg) had no effect on sIPSCs (I). Data are the mean ± SEM. Asterisks indicate a significant difference from the control (*p < 0.05) (paired *t*-test).

Given this discrepancy, the tramadol metabolite M1 was speculated to play an important role in the tramadol modulations of synaptic transmission. We determined the dosage of M1 based on a previous study regarding the time course of serum concentration of M1 after intravenous administration of tramadol [21], and found that 1 mg/kg of M1 corresponds to approximately 5 mg/kg of tramadol. Intravenous administration of M1 (1 mg/kg) decreased sEPSCs and enhanced IPSCs immediately after administration (Fig 6A and 6B). The cumulative event distribution curves obtained from the recordings in Fig 6A showed that systemic M1 (1 mg/kg) shifted the curve toward a lengthening inter-event interval and decreasing the amplitude of the EPSCs (Fig 6C). The cumulative event distribution curves obtained from the recordings in Fig 6B showed that systemic M1 shifted the cumulative event distributions of sIPSCs, to shorten the inter-event interval and increase their amplitude (Fig 6D). The suppression of sEPSCs and the enhancement of sIPSCs by systemic M1 were dose-dependent (Fig 6E).

Intravenous M1 reduced the synaptic charge transfer of sEPSCs to 64 ± 8% of the control (*n* = 5, *p* < 0.05) at 1 mg/kg and to 41 ± 9% at 3 mg/kg (*n* = 3, *p* < 0.05) and increased sIPSCs to 133 ± 10% of the control (*n* = 5, *p* < 0.05) at 1 mg and 163 ± 12% at 3 mg/kg (n = 4, *p* < 0.05). These dose-dependent effects of M1 appeared similar to those of tramadol at corresponding doses (5 and 15 mg/kg) (Fig 6E). No persistent outward current was observed after intravenous M1.

The direct actions on the spinal cord were elucidated by examining the effects of M1 superfusion. M1 was administered at a concentration of 2 μM, which might correspond to a clinically relevant concentration. The superfusion of M1 decreased the sEPSCs (synaptic charge, 74 ± 10% of control, *n* = 5, *p* < 0.05), but had no effect on sIPSCs (synaptic charge, 100 ± 3% of control, *n* = 5) (Fig 6F and 6G). The decrease in sEPSCs by M1 superfusion was abolished in the presence of naloxone (synaptic charge, 99 ± 15% of control, *n* = 5) (Fig 6H). These observations indicated that systemic M1 decreased sEPSCs via activation of the opioid receptors in the spinal cord. However, we speculated that systemic M1 enhanced the sIPSCs via supraspinal mechanisms. The involvement of supraspinal mechanisms was examined by testing the effects of systemic M1 (1 mg/kg) on the sIPSCs in spinalized rats. Systemic M1 (1 mg/kg) had no effect on sIPSCs (synaptic charge, 103 ± 5% of control, *n* = 4) (Fig 6I).

## Discussion

The present study used an *in vivo* patch-clamp technique to investigate the effects of the systemic administration of tramadol on SG neurons in the spinal dorsal horn. Systemic tramadol at doses of 5 and 15 mg/kg, but not 0.5 mg/kg, produced the following effects: a decrease in the activity of sEPSCs and an increase in the activity of sIPSCs. Both effects can inhibit the production of action potentials and attenuate the excitability of SG neurons. Increases in the nociceptive sensory threshold by systemic tramadol at 5 mg/kg, but not 0.5 mg/kg, agreed with these electrophysiological findings. Therefore, the modulations of SG neurons demonstrated in our experiments could be important antinociceptive mechanisms of systemic tramadol. Furthermore, these tramadol-induced actions on SG neurons were almost completely abolished by naloxone pretreatment, suggesting that they may be mainly mediated via the activation of μ-opioid receptors.

In clinical practice, tramadol is administered intravenously at bolus doses of approximately 100–200 mg (2–4 mg/kg). Previous clinical research demonstrated that the intravenous administration of up to 200 mg tramadol during the post-surgery period resulted in mean plasma concentrations that reached approximately 590 ng/ml, which corresponded to ~2 μM [22]. In the present study, the superfusion of 10 μM tramadol on the spinal cord had no obvious effect on the synaptic activities of SG neurons, indicating that tramadol itself had no direct effect on the spinal cord at clinically relevant concentrations. The question is then how systemic tramadol is able to modulate synaptic transmissions in the spinal cord. One possible explanation could involve an active metabolite. The tramadol metabolite M1 has an approximately ten-fold higher affinity than tramadol for μ-opioid receptors [15]. M1 appears in the plasma and reaches a maximum concentration several minutes after the intravenous administration of tramadol [21]. In our study, the decrease in sEPSCs and the increase in sIPSCs occurred after approximately 5–10 minutes and continued after the tramadol injection. M1 may therefore be speculated to modulate synaptic transmissions after tramadol administration.

The effects of intravenous M1 were examined at doses corresponding to 5 and 15 mg/kg tramadol. Systemic M1 decreased sEPSCs and increased sIPSCs in a dose-dependent manner to the same extent as seen with tramadol. These results suggested that M1 appearing after systemic administration of tramadol may be responsible for the modulation of SG neurons by systemic tramadol.

A previous study using the patch-clamp technique in spinal slice preparations showed that the μ-receptor agonist DAMGO reduced the amplitudes of evoked EPSCs and the frequencies of miniature EPSCs [23], suggesting presynaptic μ-receptor activation. The study also reported that superfused DAMGO had no effect on evoked and miniature IPSCs. In the present study, M1 superfusion at a clinically relevant concentration (2 μM) decreased sEPSCs in a naloxone-dependent manner, but had no effect on sIPSCs. Our results indicated that M1 suppressed the sEPSCs via activation of μ-receptors in the spinal cord *in vivo*.

Interestingly, in our experiments, the sIPSCs were enhanced by systemic M1 but not by M1 superfusion, which suggested that the possible involvement of the supraspinal region in the enhancement of sIPSCs by systemic M1. This possibility was examined by testing the effects of systemic M1 (1 mg/kg) on sIPSCs in spinalized rats. No obvious effect was noted on the sIPSCs in response to systemic M1, which may further support a supraspinal involvement.

Previous *in vitro* experiments using synaptosomal preparations revealed that tramadol inhibited the uptake of 5-HT and noradrenaline at clinically relevant concentrations [24, 25]. Facilitation of descending inhibitory systems by increasing extraneural noradrenaline and 5-HT_3_ is expected to participate in the antinociceptive actions of tramadol as a non-opioid mechanism. As a result, the activity of sIPSCs may be increased by activating α-1 receptors and 5-HT_3_ receptors in the presynaptic terminals of the spinal dorsal horn interneurons [26–28], while the outward currents may be induced by activating α2 receptors in SG neurons, and the activity of sEPSCs may be decreased by activating serotonin receptors and alpha-2 receptors in the terminals of primary afferent neurons [18].

Descending inhibition is also facilitated through the activation of μ-opioid receptors. Electrical stimulation of the rostral ventromedial medulla (RVM) has been shown to increase IPSCs in SG [29]. A previous study reported an abundance of μ-receptors in the RVM [30] and an injection of μ-opioid receptor agonists into the RVM produced antinociception [31]. Tramadol and M1 may facilitate descending inhibitory systems, including the RVM, via opioid and non-opioid mechanisms. Our results may implicate an involvement of descending inhibition in the modulation of SG neurons by systemic tramadol.

The superfusion experiments demonstrated that a high concentration of tramadol (100 μM) inhibited sEPSCs, induced outward currents, and enhanced sIPSCs (Fig 5), which suggested direct actions on the spinal cord. These actions may contribute to the analgesia induced by intrathecal administration of tramadol [3]. Naloxone pretreatment blocked the suppression of the sEPSCs, but not the enhancement of the sIPSCs. These results indicated that the enhancement of the sIPSCs by a high concentration of tramadol was mediated via some mechanism(s) other than μ-opioid receptor activation.

Our results clearly demonstrated the basic modulations of SG neurons by tramadol in normal rats. These modulations, however, may differ in acute and chronic pain models, where tramadol may exert additional actions. Interestingly, the mechanisms underlying the antinociceptive actions of tramadol may vary depending on the phase after chronic nerve injury. Hama et al. found that the attenuation of mechanical hypersensitivity by subcutaneous tramadol was abolished with naloxone, but not with the α2-adrenoceptor antagonist yohimbine two weeks after CCI surgery. However, analgesic effects were eventually reversed with yohimbine as well as with naloxone at 4 weeks after constriction [32]. A rat postoperative acute pain model demonstrated that the anti-hypersensitivity effects of tramadol were induced by increasing 5-HT and NA concentrations at the spinal dorsal horn, indicating the activation of descending inhibition [3]. These analgesic effects were prevented by an intrathecal pretreatment with the serotonin receptor antagonist methysergide, the noradrenaline receptor antagonist idazoxane, and naloxone. Further studies are needed in this area.

In conclusion, our *in vivo* patch-clamp study using the rat dorsal horn demonstrated that systemically administered tramadol inhibited glutamatergic transmission and enhanced GABAergic and glycinergic transmissions in SG neurons. These responses were mainly mediated by the activation of μ-opioid receptors. The tramadol metabolite M1 may be responsible for the observed effects. Inhibition of glutamatergic transmission may be mediated by the activation of μ-opioid receptors in the spinal cord and a supraspinal mechanism may be involved in the enhancement of GABAergic and glycinergic transmissions. These actions could suppress nociceptive transmission in SG neurons and contribute to the analgesic properties of tramadol.
